# Accuracy in Trace Analysis—Accomplishments, Goals, Challenges: September 28–October 1, 1987 Gaithersburg, MD

**DOI:** 10.6028/jres.093.001

**Published:** 1988-06-01

**Authors:** Richard A. Durst, Harry S. Hertz, Rance A. Velapoldi

**Affiliations:** Center for Analytical Chemistry

## Foreword

As the national reference laboratory for compositional measurements, NBS plays a major role in establishing the accuracy base for the chemical measurement system in the United States. It accomplishes this role through the issuance of Standard Reference Materials, the development of measurement methodology, and the provision of quality assurance services. In accomplishing our mission, we have a keen interest in understanding the accuracy achieved, and needed, in the ever-growing and ever-more-demanding area of trace chemical analysis. It has been almost a decade since a conference was held, at NBS, with the specific aim of reviewing the state of the art of this important measurement technology.

A decade ago, trace analysis was largely focused on environmental analyses and some health-related measurements. Since then, trace analysis has grown dramatically in number of analyses performed annually and in scope. Trace analysis has grown into a major industry in the United States, covering a broad spectrum of needs in both the private and public sectors. The applications of trace analyses have increased in areas of health and nutrition, and blossomed in the area of industrial quality assurance. The role of trace analysis in industry is certain to continue to grow as we better understand the relationships between chemical composition and product performance. Feedstock characterization, and in-line process control and quality assurance will increasingly replace after-the-fact product performance evaluation.

The use of computers in trace chemical analyses has also undergone a dramatic transformation in the past decade. Ten years ago, computers were used almost exclusively for data accumulation and evaluation. Today, computers are being used in measurement protocol design and optimization; this use will expand in the future. We will also see computers increasingly incorporated into measurement feedback loops and expert system control of measurement processes.

With the transformations in technology that have occurred in the past 10 years and with the potential for the future, it was an appropriate time to review the accomplishments of the past decade, assess the current state of knowledge in trace chemical analyses, and project future needs and directions for this area of measurement science. The papers in this issue of the *Journal of Research* provide a unique picture of a measurement science that is growing, is in great current demand, and is of vital importance to future technology and product quality in the United States and the world.

